# Benefit Assessment and Reimbursement of Digital Health Applications: Concepts for Setting Up a New System for Public Coverage

**DOI:** 10.3389/fpubh.2022.832870

**Published:** 2022-04-21

**Authors:** Hendrikje Lantzsch, Dimitra Panteli, Filippo Martino, Victor Stephani, David Seißler, Constanze Püschel, Karsten Knöppler, Reinhard Busse

**Affiliations:** ^1^Department of Health Care Management, Technische Universität Berlin, Berlin, Germany; ^2^European Observatory on Health Systems and Policies Brussels, Brussels, Belgium; ^3^German Society of Digital Medicine e.V. (DGDM), Berlin, Germany; ^4^HelloBetter—GET.ON Institut für Online Gesundheitstrainings GmbH, Berlin, Germany; ^5^fbeta GmbH, Berlin, Germany; ^6^D+B Rechtsanwälte, Berlin, Germany

**Keywords:** digital health applications, digital health technologies, medicine apps, benefit assessment, reimbursement

## Abstract

In Germany, some digital health applications (DiHA) became reimbursable through the statutory health insurance system with the adoption of the Digital Healthcare Act in 2019. Approaches and concepts for the German care context were developed in an iterative process, based on existing concepts from international experience. A DiHA categorization was developed that could be used as a basis to enable the creation of a reimbursed DiHA repository, and to derive evidence requirements for coverage and reimbursement for each DiHA. The results provide an overview of a possible classification of DiHA as well as approaches to assessment and evaluation. The structure of remuneration and pricing in connection with the formation of groups is demonstrated.

## Introduction

Digital health applications (DiHA), often also called mHealth applications, are cooperative and/or interactive applications of modern information and communication technologies, aiming to improve care and population health. They have rapidly proliferated in recent years, leading to considerable transformation in the delivery of health services (1, 2). DiHA have a wide range of different functions and are used in various areas (3). The heterogeneous structures of health systems and variability of regulations between countries mean that DiHA face different evaluation processes before inclusion into the benefits basket.

The European Commission Expert Panel on effective ways of investing in health (4) highlighted that the implementation, use, or reimbursement of DiHA should ideally be based on the evidence about their performance: DiHA should be assessed against health system objectives such as access, quality, and contribution to population health, efficiency, and equity. The proof of patient benefit is also a central element in the DiHA evaluation framework of the World Health Organization to conducting research and assessment (5). The benefit for the end user should be described and it should be demonstrated why the application is innovative, and to what extent, it is superior to the standard of care. At the same time, approaches for demonstrating the benefit of DiHA are increasingly emerging, aiming to both do justice to the particular characteristics of these health technologies and guarantee sufficient evidence to satisfy the evidence standards described above (6).

European countries apply different regulations for the payment of DiHA; they can partly be paid voluntarily by patients and individual health insurees, or individual DiHA with certain proof of patient benefit are paid obligatorily by all health insurers. Examples of countries moving in the latter direction with interesting developments in the categorization of DiHA include Belgium (7), England (8, 9), and France (10, 11). This is linked with an increased interest in reimbursement options (12–14) or assessment frameworks of DiHA (15, 16). Germany however is considered a pioneer in the European context, as a statutory reimbursement obligation has already been introduced (17). To bring DiHA into the statutory health insurance (SHI) system in Germany, the Digital Health Care Act (Digitale-Versorgung-Gesetz, DVG) was passed in November 2019 (18). The law created, among other things, the entitlement of insured persons to certain types of DiHA and broadly regulates the reimbursement claims of DiHA, and the requirements that manufacturers must fulfill to be eligible for funding. The subsequent Digital Health Applications Ordinance (Digitale Gesundheitsanwendungen-Verordnung, DiGAV) describes the procedure and specific requirements for manufacturers in detail (19).

Briefly put, to be reimbursed by SHI in Germany, a DiHA must be classified as a class I or IIa medical device according to Regulation (EU) 2017/745 on medical devices or according to Directive 93/42/EEC, serve a specific purpose, and not be used exclusively by healthcare providers/health professionals. DiHA developers must submit an application to the German Institute for Medicines and Medical Devices (*Bundesinstitut für Arzneimittel und Medizinprodukte, BfArM*), demonstrating that their application meets requirements for safety, quality, functionality, privacy, and data security. They must also demonstrate that the application has positive care effects.

It has been argued that DiHA differ substantially from both medicines and medical devices in their characteristics, and this should be considered in the design of evidence-based pathways toward reimbursement and pricing (20). Perhaps most importantly, DiHA can reflect a care process rather than a single product. At the same time, DiHA compete for the same pot of funds as other health interventions, and the principles underpinning decisions on their reimbursement should be comparable. This article addresses the question of how these supposedly contradictory statements can be united. In the following, approaches developed in the “I.DiGA project” (see Methods Section, next) for a benefit assessment and pricing mechanism for DiHA will be presented. While the project focused on the German context, the approaches described in the following may be useful for other countries considering setting up similar systems.

## Methods

Parallel to the German “Digital Health Care” (DVG) legislative process, the “I.DiGA project,” funded by the Federal Ministry of Health, developed approaches for the categorization, evaluation, and pricing/reimbursement of DiHA. The project took place in parallel with the preparation, discussion, and adoption of the DVG and related regulatory documents, and its results served as one of the bases that informed this process. The project had an agile design: contents were developed drawing on existing international frameworks and initiatives, literature research, and expert interviews. These contents were then discussed in seven expert workshops involving a broad range of stakeholders to identify areas in need of refinement and additional analysis, and possible solutions were developed. There were three main areas of investigation: (a) the development of a conceptual model for a DiHA directory that could serve reimbursement purposes; (b) the development of a framework for the evidence-based evaluation of DiHA, encompassing process and methodological requirements; and (c) the development of a remuneration system for DiHA, including a pricing approach.

## Results

### Developing a “Positive List” for DiHA: Proposal for a DiHA Directory

Given the heterogeneity of DiHA, as well as the availability of several DiHA serving the same purpose, it was necessary to create a taxonomy that would facilitate reimbursement, pricing, and prescribing decisions. We developed a categorization that could serve as the basis for a directory with a triple functionality: grouping DiHA in a meaningful way to enable the determination of the type of evidence required for reimbursability; to identify comparators, against which additional benefits can be demonstrated; and to enable DiHA comparisons for health professionals looking to choose which alternative to prescribe. We proposed a “directory for digital health applications” that groups DiHA according to the following attributes: (i) application area, (ii) target group, (iii) function, and (iv) user. It is listed in Table 1.

**Table 1:** Proposal for DiHA directory with criterion values and correspondence to evidence requirement levels (blue shading).

**i. Scope (according to ICD-10)**	**ii. Target group**	**iii. Function of the DiHA**		**iv. User**	**Seq. No**.
...	**0** Healthy without known risk factors	**0** Documentation		**1** Only patients or relatives	001
					002
					003
					…
**Exx** Endocrine, nutritional and metabolic diseases	**1** Healthy with risk factors	**1** Information		**2** Patients and Service Providers	001
					002
					003
					…
**Fxx** Mental and behavioral disorders	**2** Acutely ill, not life-threatening	**2** Prevention, prediction, prognosis		**3** Service providers only	…
**Hxx** Diseases of the eye and ear	**3** Chronically ill, stable state of health	**3** Examination of a physiological or pathological process or condition			…
**Ixx** Diseases of the circulatory system	**4** Highly vulnerable/ unstable health status	Detection (diagnosis)	**4** Diagnosis		…
**Oxx** Pregnancy, Birth and Postpartum		Supervision (monitoring)	**5** Simple monitoring		…
			**6** Complex monitoring		
…		Treatment (therapy)	**7** Indirect intervention (self-management)		…
			**8** Direct intervention (change in health conditions)		

Light blue filling = Low requirement level; Medium blue filling = Medium requirement level; Dark blue filling = Dark requirement level.

The application area (i) is derived from the 3-digit ICD 10 code of the disease for which the DiHA is to be used according to its certification and this, together with the function, reflects the intended purpose of the DiHA as a medical device. The target group (ii) complements the scope of application and reflects the disease severity or vulnerability of the patients who are to receive the DiHA. The classification varies because the evidence requirements for healthy people may be lower than for highly vulnerable people. The function (iii) is divided into detection, monitoring, and treatment (including alleviation), which are further differentiated according to their exact functionality into (4) Diagnosis, (5) Simple Monitoring, (6) Complex Monitoring, (7) Indirect Intervention, and (8) Direct Intervention. Under “function,” Categories 0–3 represent possible functionalities of DiHA as medical devices according to Regulation (EU) 2017/745, which are, however, not among those meeting the requirements of the 2020 iteration of the DVG for prescribability (these were included in the model to ensure its sustained usability should the scope of the legal framework be expanded in future). The user (iv) of a DiHA may not only determine the risk associated with its use, but also influence the evaluation design and the potential access and remuneration pathways. For the user, a distinction is made as to whether the DiHA is used (1) by the patient or a relative only, or (2) by the patient and healthcare provider. Category (3) is reserved for DiHA that are used exclusively by health professionals and are therefore not covered by the DVG (illustrated here for the same reason as Categories 0–3 under “function”).

For each criterion, a DiHA included in the SHI reimbursement list would be assigned the matching digits depending on taxonomic position, and those would be sequentially merged into a 6-digit number. Additional digits are added at the end to provide a serial number for each DiHA (product) within a single taxonomic position (Table 1).

### Evidence Requirements for the Reimburseability of DiHA

The DiHA classification system described above can serve inter alia to link the identified DiHA groups to the level of evidence required for the DiHA to demonstrate positive care effects and qualify for reimbursement. For reasons of usability, the I.DiGA project proposed a simplified distinction between three evidence requirement levels: low, medium, or high. The requirement level for each DiHA group in the matrix is determined by the two attributes “target group” and “function.” The darker the shade of the cell in Table 1, the more rigorous the study design of the evidence required for reimbursability. The final level of evidentiary requirements for each group of DiHA is determined by the highest level assigned to one of the two categories (e.g., a direct intervention for stable chronic patients would fall into the “high” evidence category, determined by the high assignment for DiHA with this function).

To further specify the acceptable evidence per requirement level, considerations from existing frameworks were combined (5, 8, 10). Three fundamental elements of study design were chosen to provide the necessary foundation and retain simplicity. Expectations on study design increase with the level but the comparison against a control group was considered essential for all levels. Only DiHA in the low requirement category can potentially rely on study designs wherein participants form their own comparison group (before/after designs) to qualify for reimbursability. A medium requirement level means it is sufficient to present results from (quasi-)experimental studies with a control group. While the randomized allocation to the intervention and control group is not a prerequisite, all relevant confounding variables should be identified and considered to the maximum possible extent. The analysis must follow the “Intention to treat” (ITT) principle. When DiHA fall into a high requirement category, a randomized controlled study with an analysis following the ITT principle is required (Table 2).

**Table 2:** Study design characteristics per requirement level.

	**Low**	** Middle**	**High**
Control group	X (before-after)	X	X
Intention-to-treat evaluation		X	X
Randomized group allocation			X

Light blue filling = Low requirement level;

Medium blue filling = Medium requirement level;

Dark blue filling = Dark requirement level.

#### Acceptable Outcomes for Demonstrating the Effects of DiHA

Due to their nature, DiHA can have health effects and other effects. Taking the overall aim of improving patient-relevant outcomes, the project relied on the assumption that for the overall effect of DiHA to be positive, either the health effects must be better with the DiHA than without the DiHA, or the other effects must be better if the health effects are the same (i.e., health effects should not be jeopardized even if other effects are better; the general interpretation of the DVG in Germany deviates from this position, see Section Discussion).

For demonstrating the health effects of DiHA, end points that reflect patient-relevant benefits in the form of an improvement in health status, shortening of disease duration, prolongation of life, reduction of side effects, or improvement in quality of life are ideal. On the other hand, typical surrogate parameters can also be acceptable under certain circumstances, as can other intermediate end points such as medication adherence; provided that their correlation with patient-relevant end points is established (i.e., they have been validated).

Similar to the logic for determining the evidence requirement levels described above, acceptable end points depend on the type of DiHA as determined by its function and target group. Table 3 brings together these two attributes with the corresponding level of required evidence and the respective acceptable end points for determining health effects. For DiHA that make a diagnosis, take over complex monitoring, or represent a direct intervention to change health status, relevant health-related end points should be investigated with robust study designs. Surrogate parameters can be used as end points for these DiHA only if non-vulnerable target groups are involved. For the DiHA that correspond to indirect interventions or simple monitoring, the spectrum of health-related end points can be expanded to include other intermediate outcomes.

**Table 3:** Matrix for determining the requirement level and acceptable end points for health effects.

**Categorization regarding “target group”**	**Categorization regarding “function”**										
	4 Diagnosis										
	8 Direct intervention										
	6 Complex monitoring			7 Indirect intervention				5 Simple monitoring			
4 Highly vulnerable and/or unstable health status	**High**		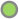	**High**		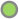		**High**		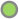	
2 acutely ill, not life-threatening/ 3 chronically stable	**High**			**Medium**				**Medium**			
1 healthy with risk factors	**High**	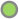	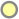	**Medium**	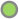	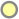	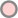	**Low**	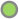	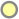	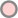
		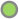	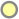		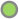	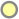	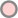		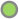	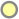	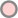
Acceptable endpoints for health care effects	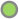 Patient-relevant benefit			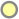 Efficacy (surrogate parameters if applicable)				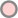 other intermediate outcomes, e.g. adherence			

Light blue filling = Low requirement level; Medium blue filling = Medium requirement level; Dark blue filling = Dark requirement level.

As DiHA often reorganize whole processes of care, non-health outcomes can be used to determine reimbursement, provided that the health effects are not compromised. The field of health technology assessment (HTA) has long considered the potential implications of the application of health technologies across a range of domains, and these can also be used to derive outcome categories here (21). Specifically, organizational, social/ethical, and economic aspects were considered particularly relevant for determining a DiHA's eligibility for reimbursement. Building on the EUnetHTA Core Model®, core aspects with associated contextual questions can help identify possible outcome areas and target variables (see Appendixes 1–5 in Supplementary Material). As with health effects, the evaluation of other care effects of DiHA should be based on a comparative analysis, if they are to *improve* patient outcomes. While established outcomes for the measurement of health and economic effects are available, this is largely not the case for organizational and social/ethical effects. How these can be measured depends on function and target group and cannot be determined uniformly.

#### Relationship Between Health Effects and Other Care Effects

As described earlier, the I.DiGA project worked on the assumption that other care effects should be used to determine reimbursability only if sufficient evidence has shown that health effects are superior, or at least not inferior to those of the appropriate comparator. Drawing on similar considerations in France (10), the matrix in Table 4 was designed to visualize the interplay between health effects, other care effects, and overall reimbursability. If neither the health effects nor the other care effects are superior to a corresponding comparison, eligibility for reimbursement should not be considered given.

**Table 4:** Relationship between health effects and other care effects.

**Compared to an appropriate comparator**	**Health effects regarding the DiHA's function/purpose**	**Other care effects (organizational, social/ethical, economic)**	**Overall evaluation (eligibility for reimbursement)**
Superior	+	+	+
	+	0	+
	+	–	+
Comparable	0	+	+
	0	0	–
	0	–	–
Worse	–	Not relevant	–

### Pricing and Remuneration Systems of DiHA

#### Principle of Price Determination for DiHA

Based on the usual pricing methods applied or discussed for health technologies, the amount public payers are to spend on DiHA qualifying for reimbursement can be based on (a) the extent of the positive effect proved in studies and/or (b) development and production costs and/or (c) a price comparison (national/European consumer prices or European reimbursement prices). Depending on health system goals and payer criteria, price negotiations can consider only positive health effects, or also other care effects such as increased efficiency. Based on any one or a combination of these three components, an initial base amount to be paid for a DiHA can be calculated, potentially complemented by performance-based remuneration components for actual positive effects achieved by patients using the DiHA. DiHA utilization data or patient-reported outcome and/or experience measures can be used to determine such components.

#### Considerations on a Remuneration Model

The proposed logic for a remuneration and pricing model builds on the previous ideas of clustering DiHA based on indication, function, and target group and requiring comparative evidence before they can qualify for reimbursement. For DiHA with comparable measured effects and underlying study quality, prices should also be comparable. A new DiHA would only be eligible for a higher price if it were superior in term of positive effects and/or was supported by methodologically better studies than already reimbursed DiHA in the same group. An additional dimension that may contribute to a higher price within a DiHA group concerns the comparator in the submitted studies: a new DiHA that can show benefit compared to existing DiHA would receive a higher price than DiHA that have shown benefit compared only with no DiHA use. If the three criteria positive effects (by type and extent), quality of evidence (including overall study design and quality of individual studies), and comparator are combined, a classification leading to different price tiers can be created (Table 5).

**Table 5:** Possible price tiers based on the criteria of study results, quality of evidence, and comparator.

**Study design**	**Study quality**	**Type of positive effect**	**Extent of the (additional) effect**	**Comparator**	**Price tier**
Not appropriate*	Irrelevant				Not reimb.
Appropriate*	Not according to best practice	Health effect OR other care effect only	Irrelevant		Not reimb.
		Health effect AND other care effect	Irrelevant		Tier 1
	According to best practice	Other care effect (health comparable)	Irrelevant		Tier 1
		Health effect (and possibly other care effect as well)	Small	No DIHA or DiHA tier 1	Tier 1
				DiHA tier 2	Tier 2
			Considerable/substantial	No DiHA	Tier 2
				DiHA tier 1/2	Tier 3
				DiHA tier 3	Tier 4

* *Low/middle/high depending on the requirement according to Table 2*.

## Discussion and Policy Implications

The aim of the I.DiGA project was to provide a basis for a workable system of implementing DiHA in the public reimbursement system, particularly for the German context. With the DVG, Germany is considered a pioneer, where patient-facing DiHA are now reimbursable by the SHI system. However, the project aimed at providing conceptual guidance usable beyond the context of the DVG as passed in 2020, regarding, for instance, an expansion in the scope of regulated DiHA. It should be helpful for relevant stakeholders thinking about how to set up a public system for DiHA access in any country.

Several countries have developed or are developing approaches to better understand and evaluate DiHA for health system use; however, these are at different stages of development and clearly dependent on the country's health system set-up and coverage decision-making processes so far (4, 22, 23). Concerns regarding privacy, efficacy, usability, and implementation of DiHA are common characteristics of all these initiatives. These could potentially be alleviated by clear evaluation guidelines from the relevant decision-makers, as they have now been developed in Germany (24).

The system to determine the reimbursability of DiHA presented in this paper has similarities with other approaches, such as the Belgian validation pyramid (7), but also important differences, such as the categorization based on target group and function. The categorization matrix is more granular than existing alternatives for a formal app repository, such as the National Health Service (NHS) App (9). Regarding evidentiary requirements and related pricing options, our model draws on the NICE framework from England (8), and the Medical Device and Health Technology Evaluation Committee (CNEDiMTS) framework (10), where, however, the number of evidence levels and the level of detail of reimbursement situations differ.

A systematic review of evaluation frameworks for mobile health applications found that the majority of the identified frameworks that examined safety—only a few assessed potential harm and only one explicitly considered a comparison group (25). However, it has been recognized that health systems must provide adequate approaches for DiHA evaluation and reimbursement (5, 26), and HTA can provide a methodological basis (27). To make informed decisions about the reimbursement, prescription and use of DiHA, stakeholders, clinicians, and users need guidance that adapts to diverse local requirements, standards, and current content (28). To create a standard set of requirements, state regulatory agencies should collaborate with clinical stakeholders (22). Assessment tools for DiHA that aim to measure risks and benefits are proliferating (29). Methodological considerations for evaluating DiHA are increasingly developed, for instance, primarily for specific DiHA groups [e.g., for mental health, see (30), or with a more comprehensive scope (31)].

The work in the I.DiGA project confirmed the national and international relevance of the topic. Even though there are more and more approaches toward DiHA evaluation and reimbursement decisions, none have really been tested yet in the real world. Consequently, the implementation of DiHA reimbursement processes should be closely monitored and evaluated, and shared in international dialogue. The concepts developed in the I.DiGA project can provide an initial basis but will likely need to be adapted once the implementation of current provisions in Germany and other countries has been evaluated. The exchange of knowledge and experience at European level could contribute to the improvement and expansion of relevant assessment frameworks; existing initiatives should be supported, and funding should be made available for implementation research.

The study was developed based on the regulations and particularities of the German health system; this may be considered a limitation, or at least a challenge for transferability. However, even if other countries have different starting points, our considerations can be adopted to some extent. Another limitation of our work pertains to the selective consideration of existing literature and country examples for the evaluation of DiHA, as no systematic search was carried out. A number of new publications may have become available since this work was concluded. The insights in this paper are mainly conceptual in nature and have not been validated yet; however, they partially draw on existing and tested regulatory levers for other health technologies in the German health system.

## Conclusion

The I.DiGA project developed concepts to categorize, evaluate, and price DiHA for reimbursement. These can be used individually or in combination to inform the formalization of any DiHA reimbursement system. Fundamentally, DiHA that want to be covered by statutory health systems need to provide robust evidence of patient benefit, which can be used to determine reimbursement eligibility and price. As other European countries are also increasingly developing approaches for categorizing or reimbursing DiHA, cross-border cooperation for the development of efficient evaluation methods which consider the particularities of DiHA is indicated, and has the potential of expanding reimbursement opportunities for developers.

## Author Contributions

HL, DP, and RB wrote the manuscript. All authors conceived the contents of the project and revised the manuscript. All authors contributed to the article and approved the submitted version.

## Funding

The I.DiGA project was funded by the German Federal Ministry of Health (Grant Reference: ZMVI1-2519FSB800).

## Conflict of Interest

VS was employed by HelloBetter—GET.ON Institut für Online Gesundheitstrainings GmbH. DS and KK were employed by fbeta GmbH. CP was employed by D+B Rechtsanwälte. The remaining authors declare that the research was conducted in the absence of any commercial or financial relationships that could be construed as a potential conflict of interest.

## Publisher's Note

All claims expressed in this article are solely those of the authors and do not necessarily represent those of their affiliated organizations, or those of the publisher, the editors and the reviewers. Any product that may be evaluated in this article, or claim that may be made by its manufacturer, is not guaranteed or endorsed by the publisher.
