# Supplementary material for: Benefit Assessment and Reimbursement of Digital Health Applications: Concepts for Setting Up a New System for Public Coverage
Source: Front Public Health. 2022 Apr 21;10:832870. doi: 10.3389/fpubh.2022.832870 (PMC9068958; doi:10.3389/fpubh.2022.832870)
Supplement: Supplementary file 1 [file Data_Sheet_1.docx]

Supplementary Material

# Supplementary Tables

*Appendix 1: Core aspects and contextual questions in the domain of organisational supply effects*

| Key aspects | Context questions |
| --- | --- |
| Treatment process | How does DiGA affect previous work and supply processes? |
|  | How does the use of DiGA change the care process from the patient's perspective? |
|  | Does the DiGA have an influence on the communication and cooperation between different actors? |
|  | Does the DiGA change the participation of individual actors (e.g. nurses, relatives) in the treatment process? |
|  | What is the effect of DiGA on the quality assurance of the treatment (also across all LE)? |
| Supply­ structure | What influence does DiGA have on access to health care? |
|  | Does the use of DiGA change the location of medical care? |

*Appendix 2: Exemplary outcomes and target figures in the domain of organisational care effects*

| **Outcome** | **Example target value** | **Possible survey method** |
| --- | --- | --- |
| Coordination of treatment procedures (e.g. waiting times, service provider/patient contacts, treatment management, insights into treatment results) | Waiting times of patients at the service provider | Questionnaire |
|  | Service provider/patient contact | Supply data |
|  | Low-threshold exchange of treatment-relevant data between service provider and patient | Questionnaire |
| Treatment in line with guidelines | Implementation of the recommended guideline in diagnosis and therapy by patients | Questionnaire |
| Participation of relatives | Time and effort for caring relatives | Questionnaire; focus group |
| Facilitating access to care | Availability of service providers in rural areas (access to specialist medical care) | Questionnaire |
| … | … | … |

*Appendix 3: Core aspects and contextual questions in the domain of social/ethical care effects*

| Key aspects | Context questions |
| --- | --- |
| aspects of patients and social groups/  Communication | To what extent does the DiGA make it possible to fulfil the expectations and wishes of patients? |
|  | What are the experiences with the state of health or disease and DiGA to live? |
|  | Are caring relatives relieved by the use of the DiGA? |
|  | Does DiGA create better access for patient groups who currently do not have good access to available therapies? |
|  | Does the DiGA allow better information about treatment options for patients? |
|  | Does the DiGA allow the patients to be involved in treatment decisions? |
|  | Does DiGA increase the health competence of patients? |
| Benefit/risk profile | What is the perceived benefit/risk of DiGA from the patients' perspective? |

*Appendix 4: Exemplary outcomes and target figures in the domain of social/ethical care effects*

| **Outcome** | **Example target value** | **Possible survey method** |
| --- | --- | --- |
| Adherence* | Achievement of (participative) therapy goals agreed between the service provider and patient | Questionnaire |
| Inequality | Access to hard-to-reach patient groups (e.g. due to socio-economic status) | Questionnaire |
| Health literacy | Improvement of individual health literacy | Questionnaire |
| Patient sovereignty | Being informed about further treatment options (patient's knowledge about treatment alternatives) | Questionnaire |
|  | Shared Decision Making  (involvement of patients in treatment decisions) | Questionnaire |
| Coping with illness-related difficulties in everyday life | Early warning of seizures (e.g. epilepsy) | Questionnaire |
|  | Participation in everyday or social activities | questionnaire, focus group |
| Satisfaction, psychological well-being and need orientation | satisfaction with the supply | Questionnaire |
|  | Well-being and emotional state | Questionnaire |
|  | (Holistic) satisfaction of basic physical and psychological needs | Questionnaire |
| Acceptance | Patient acceptance of the treatment | Questionnaire |
| Self-motivation | Development of individual approaches for dealing with the disease | questionnaire, focus group |
| Reduction of therapy-related expenses and burdens for relatives | Stress load and stress management of the caring relatives | Questionnaire |
| ... | … | … |

**A special case is the outcome "adherence". In the logic of the I.DiGA project, this can serve on the one hand as a borderline case in proving a positive, health care effect. On the other hand, adherence can also represent a social/ethical supply effect.*

*Appendix 5: Core aspects and contextual questions in the domain of economic supply effects*

| Key aspects | Context questions |
| --- | --- |
| Resource use and cost/outcomes ratio | What types of resources are used in the provision of the DiGA and its comparators? |
|  | How many units of these resources are consumed in providing the evaluated DiGA and its comparators and how are these units valued in monetary terms? |
|  | How does DiGA change the need for other technologies and the use of other resources? |
|  | What are the possible budgetary implications of the technologies to be compared? |
|  | What are the estimated differences in the cost/output ratio between the DiGA and its comparators? |
|  | What types of resources are used in the provision of the DiGA and its comparators? |
